# A fused biometrics information graph convolutional neural network for effective classification of patellofemoral pain syndrome

**DOI:** 10.3389/fnins.2022.976249

**Published:** 2022-07-29

**Authors:** Baoping Xiong, Yaozong OuYang, Yiran Chang, Guoju Mao, Min Du, Bijing Liu, Yong Xu

**Affiliations:** ^1^School of Computer Science and Mathematics, Fujian University of Technology, Fuzhou, China; ^2^Fujian Provincial Key Laboratory of Big Data Mining and Applications, Fujian University of Technology, Fuzhou, China; ^3^Fujian Provincial Key Laboratory of Eco-Industrial Green Technology, Wuyi University, Wuyishan, China; ^4^State Grid Electric Power Research Institute, Beijing, China

**Keywords:** patellofemoral pain syndrome, graph convolutional neural network, fuse biometrics, semi-supervised classification, auxiliary diagnostic

## Abstract

Patellofemoral pain syndrome (PFPS) is a common, yet misunderstood, knee pathology. Early accurate diagnosis can help avoid the deterioration of the disease. However, the existing intelligent auxiliary diagnosis methods of PFPS mainly focused on the biosignal of individuals but neglected the common biometrics of patients. In this paper, we propose a PFPS classification method based on the fused biometrics information Graph Convolution Neural Networks (FBI-GCN) which focuses on both the biosignal information of individuals and the common characteristics of patients. The method first constructs a graph which uses each subject as a node and fuses the biometrics information (demographics and gait biosignal) of different subjects as edges. Then, the graph and node information [biosignal information, including the joint kinematics and surface electromyography (sEMG)] are used as the inputs to the GCN for diagnosis and classification of PFPS. The method is tested on a public dataset which contain walking and running data from 26 PFPS patients and 15 pain-free controls. The results suggest that our method can classify PFPS and pain-free with higher accuracy (mean accuracy = 0.8531 ± 0.047) than other methods with the biosignal information of individuals as input (mean accuracy = 0.813 ± 0.048). After optimal selection of input variables, the highest classification accuracy (mean accuracy = 0.9245 ± 0.034) can be obtained, and a high accuracy can still be obtained with a 40% reduction in test variables (mean accuracy = 0.8802 ± 0.035). Accordingly, the method effectively reflects the association between subjects, provides a simple and effective aid for physicians to diagnose PFPS, and gives new ideas for studying and validating risk factors related to PFPS.

## Introduction

Patellofemoral pain syndrome (PFPS) is a common chronic knee injury (Boling et al., 2009), which typically presents as diffuse anterior knee pain (Crossley et al., 2016). According to studies, the annual prevalence for PFPS in the general population is 22.7 % and in adolescents is 28.9 % (Smith et al., 2018). Sociodemographic characteristics [e.g., height, weight, and body mass index (BMI)] do not have a significant impact on the incidence of PFPS (Collins et al., 2010). However, females have a higher incidence of PFPS compared with males (Boling et al., 2010). Daily physical activities with knees bending (e.g., climbing stairs, squatting, running, jumping, sitting) can exacerbate the pain that causes limitations to daily activities (Halabchi et al., 2013). Increasing evidences suggest that it is a recalcitrant condition that can persist for many years (Crossley et al., 2016). In addition, PFPS patients have a higher risk of osteoarthritis (OA) (Thomas et al., 2010).

Early accurate diagnosis of PFPS helps avoid disease progression (Ferrari et al., 2014). But there is still a lack of consensus on the exact pathological mechanism of PFPS, as its etiology is multifactorial (Powers et al., 2012). Clinical examination is the cornerstone to diagnose PFPS, and the best available test is anterior knee pain elicited during a squatting maneuver. Tenderness on palpation of the patellar edges, patellar grinding, and apprehension tests are also capable of detecting (Crossley et al., 2016). The clinical diagnosis of PFPS usually uses an exclusion method without the need of any further imaging studies (Nuttall and Winters, 2015). The currently proposed pathomechanical model suggested that PFPS is associated with abnormal loading of the patellofemoral joint (elevated joint stress) (Powers et al., 2017). Describing the causal relationship between tissue stress and pain is difficult because these variables cannot be measured directly *in vivo*. To solve the problem, human kinematics and surface electromyography (sEMG) can be used to set up a corresponding musculoskeletal model to estimate the joint moment (Buchanan et al., 2004). Since the coordination mechanism of the human nerve, muscle, and skeletal system are unknown, such models will inevitably be faced with some uncertainties such as inaccuracy or computational complexity, which is not conducive to the application in clinical testing.

Previous studies have shown that data-driven machine learning has good system representation and individual adaptability when the principles of the system are unclear or unknown (Zeng et al., 2016). Recently, machine learning has been increasingly used in biomedical sciences, such as joint moment prediction (Myer et al., 2014; Xiong et al., 2019) and disease diagnosis (Shi et al., 2021a,b) etc. These methods mainly focus on biological signal information of individuals but neglect the common characteristics of patients. In practical situations, however, it is beneficial to consider the relationship among patients as it facilitates the analysis and study of patient groups with similar symptoms (Ghorbani et al., 2021).

As a graph-based neural network model, GCN can focus on both individual characteristics and common features (Ahmedt-Aristizabal et al., 2021; Li et al., 2022b). The graphs provide an intuitive way to represent individuals (as nodes) and the common features of patients (as edges) (Zhou et al., 2020). Currently, GCN has been successfully applied in the areas of disease diagnosis (Parisot et al., 2018), emotion recognition (Song et al., 2020), and heart abnormality detection (Wang et al., 2020), etc. In addition, GCN is also a semi-supervised learning method, which uses samples without class labels to train the samples with class labels, which makes it an attractive approach toward addressing the problem of data shortage (Oliver et al., 2018).

**FIGURE 1:**
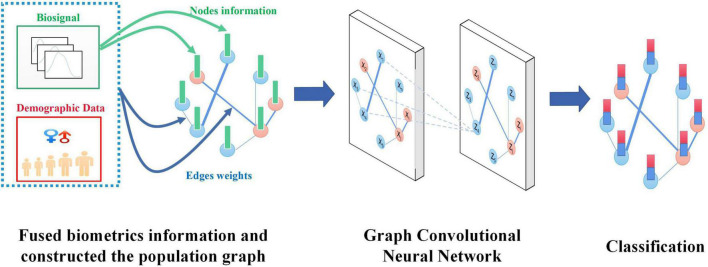
An overview of the FBI-GCN model.

In this work, we propose a method based on the fused biometrics information Graph Convolution Neural Networks (FBI-GCN) to assist the diagnosis PFPS. A population graph was constructed in which the similarity of test subject characteristics (sEMG and joint kinematics) and demographic information were fused to represent the degree of association between different subjects. The FBI-GCN was used to focus on both individual characteristics (inputs of nodes) and common features, and the classes of unlabeled nodes were inferred by training the model. The model was first tested on 41 subjects using the public datasets of PFPS to explore the best strategy for constructing the population graph. Then the impact of the choice of each part of the model on the classification performance was examined. The overview of FBI-GCN is shown in Figure 1. Finally, the model was used to validate some of the current clinical findings on the PFPS, and optimized the selection of input variables according to expert knowledge, which could still obtain a high diagnostic accuracy while greatly reducing the cost of data collection. Experiment results showed that recognizing the correct clinical knowledge can significantly improve the classification performance of the model.

The main contributions of this research include:

(1) The method of graph structure was used to construct the relationship between individual characteristics and group common characteristics between subjects. At the same time a semi-supervised, GCN-based PFPS auxiliary diagnosis method (FBI-GCN) was designed, which is an end-to-end model with a relatively simple structure design to achieve a high detection accuracy in public datasets.
(2) The model was validated by some of the current clinical findings on PFPS, which is verified by experiments to show that accurate and effective common features (gender information) can significantly improve diagnostic accuracy.
(3) Feature optimization through expert knowledge remains high diagnostic accuracy while reducing experimental costs.

## Materials and methods

### Datasets and pre-processing

The experimental data used in this paper was downloaded from the public datasets published on the website,^1^ which was accessed on 18 October 2020. This dataset provides lower limb kinematics and sEMG of 26 patients (16 females and 10 males) with patellofemoral pain and 15 pain-free control subjects (8 females and 7 males) during walking and running.

The biometric information available in this dataset includes demographics and gait biosignal (sEMG and joint kinematics) of subjects. The lower limb kinematics involves the hip, knee, and ankle flexions. The muscles that are used to collect sEMG signals include the rectus femoris (RF), vastus medialis (VASMED), vastus lateralis (VASLAT), medial hamstrings (SEMIMEM), lateral hamstrings (BIFEMLH), medial gastrocnemius (MEDGAS) and lateral gastrocnemius (LATGAS). The sEMG signals were low-pass filtered using a zero-lag fourth-order Butterworth filter with a cut-off frequency of 15 Hz. Details about the collection process of the entire dataset can be found in reference (Besier et al., 2009). A summary of the subjects is shown in Table 1.

**TABLE 1:** Mean ± standard height and mass of the subjects.

	PFPS		Control	
	Males (*n* = 10)	Females (*n* = 16)	Males (*n* = 7)	Females (*n* = 8)
Height (m)	1.78 ± 0.08	1.68 ± 0.06	1.80 ± 0.05	1.66 ± 0.05
Mass (kg)	73.5 ± 15.7	62.7 ± 10.0	73.4 ± 18.1	58.3 ± 4.6
Body mass index	22.60 ± 2.80	23.03 ± 3.20	21.21 ± 1.87	21.89 ± 2.00

Since the inconsistent feature scales (measurement units) of the data are not conducive to model convergence, normalization of the raw data is required. The Z-score is used to normalize the data:


(1) $$X_{i}^{\prime }=\frac{X_{i}-\mu_{i}}{\sigma_{i}}$$

where *X*_*i*_ is the node feature vector, μ_*i*_ and σ_*i*_ represent the expected value and standard deviation of *X*_*i*_.

### Construction of the population graph

The input of GCN requires a graph in addition to feature vectors of nodes. Improperly constructed graph (the graph that does not properly represent similar relationships between subjects) will have a negative impact on the classification performance. The population was represented as a weighted graph *G* = (*V, E, Ã*), where *V*denotes the nodes of the graph; *E*denotes the set of edges connecting these nodes; *Ã* denotes the weighted adjacency matrix of the graph. The adjacency matrix describes the connectivity between any two nodes, where the importance of the connection between the node *i* and node *j* is represented by *a*_*ij*_.

The goal is to assign a label *l* ∈ {0, 1} to each graph node that describes whether the subject is pain (*l = 1*) or control (*l = 0*). Previous studies have shown that demographic data can provide useful information to mine the association with subjects (Zheng et al., 2022). Here the demographic information (gender, height, weight, etc.) is defined as *M* = {*M*_*h*_}, *H* is the number of demographic data types to be calculated. The GCN modeling method is referenced to that proposed in Parisot et al. (2018) and the adjacency matrix *W*of a graph is calculated by equation (2).


(2) $$\tilde{A}\left(i,j\right)=Sim\left(V_{i},V_{j}\right)\sum_{h=1}^{H}\gamma \left[M_{h}\left(i\right),M_{h}\left(j\right)\right]$$

where γ is a measure of the distance between demographic information, and *Sim*(*V*_*i*_, *V*_*j*_)represents the edge weight constructed based on the feature similarity vector between node *i* and node *j*. The threshold Gaussian similarity kernel function is used in this paper, which is worked out by equation (3).


(3) $$Sim\left(V_{i},V_{j}\right)=\left\{ \begin{array}{c} \exp\left(-\frac{{\left[\rho \left(X_{i},X_{j}\right)\right]}^{2}}{2\theta^{2}}\right),if\rho \left(X_{i},X_{j}\right)\geq \tau \\ 0,otherwise \end{array}\right.$$

where ρ(*X*_*i*_, *X*_*j*_) is the similarity between node feature vectors *X*_*i*_ and *X*_*j*_, τ is the threshold, and θ represents the width of the kernel. Ideally, subjects belonging to the same category (control or pain) should have a greater similarity weight than those belonging to different categories. In order to ensure the sparsity of the adjacency matrix and make it easier to separate the nodes, a threshold τ is set. When the similarity of two nodes is greater than the threshold, the two nodes are considered connected. This method was inspired by Zeng et al. (2022).

The Kronecker delta function δ (Parisot et al., 2018) was used for function γ. The value of γis 1 if the two variables are equal, otherwise it is 0. But in the case of real numbers, such as weight, height, etc., they cannot be exactly equal. Therefore, we define it as a unit step function with respect to a threshold by equation (4):


(4) $$\gamma \left(M_{h}\left(i\right),M_{h}\left(j\right)\right)=\left\{ \begin{array}{c} 1,if\left|M_{h}\left(i\right)-M_{h}\left(j\right)\right|\leq \lambda \\ 0,otherwise \end{array}\right.$$

where λ is the threshold. The value of γ can be adjusted based on some prior knowledge. For example, females are susceptible to PFPS at a 2–3 times higher rate than males (Boling et al., 2010). It means that females should have a higher influence on the edge weight of the graph, so we define the function γ as equation (5):


(5) $$\gamma \left(M_{h}\left(i\right),M_{h}\left(j\right)\right)=\left\{ \begin{array}{c} \lambda_{1},if\;M_{h}\left(i\right)=M_{h}\left(j\right)=Male\\ \lambda_{2},if\;M_{h}\left(i\right)=M_{h}\left(j\right)=Female\\ 0,otherwise \end{array}\right.$$

where λ_*1*_ and λ_*2*_ are adjustable constants and λ_*1*_<λ_*2*_. For other demographic information, piecewise functions can also be established based on expert knowledge, and the modeling method can refer to equation (5).

### Spectral-GCN

The structure of the graph is usually complex and irregular, where each node carries its own local features and does not have general translation invariance. The traditional convolutional neural network (CNN) is not suitable for such classification problems. Therefore graph-oriented convolution operations need to be introduced, and spectral-GCN is one of the applicable methods which convolves on graphs (Kipf and Welling, 2016).

The GCN model used in this paper follows that proposed by Kipf and Welling (2016) because it simplified the graph convolution layer using the following equation:


(6) $$Z={\tilde{D}}^{-\frac{1}{2}}\tilde{A}{\tilde{D}}^{-\frac{1}{2}}X\Theta$$

where *Ã* = *A* + *I_N_*, *I*_*N*_ denotes the identity matrix, *A* denotes the adjacency matrix, ${\tilde{D}}_{ii}=\sum_{j}{\tilde{A}}_{ij}$, *X* ∈ *R*^*N*×*C*^ is the eigenvector of dimension *C* in each node, Θ ∈ *R*^*C*×*F*^ is the parameter matrix of the filter, *Z* ∈ *R*^*N*×*F*^ is the convolution signal matrix, and *F*is the number of filters.

**FIGURE 2:**
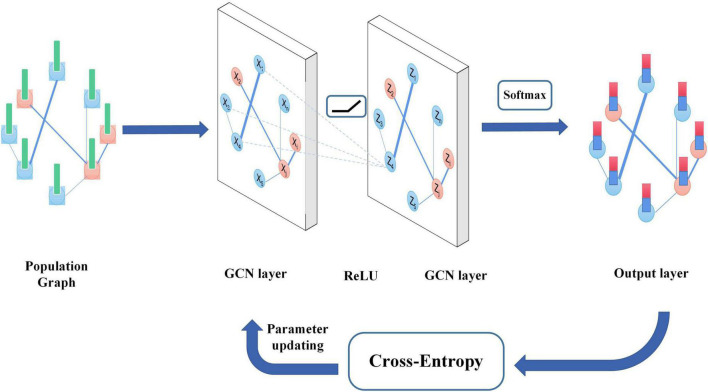
The algorithm overview of GCN.

The algorithm overview is shown in Figure 2. The FBI-GCN is relatively simple, which is designed as a two-layer GCN with each convolutional layer followed by a rectified linear unit (ReLU) activation function for the introduction of non-linearity. The output layer is followed by a softmax activation function and the maximum value of the output is assigned labels to unlabeled nodes. The forward phase is expressed as follows:


(7) $$Z=f\left(X,A\right)=\text{softmax}\left(\tilde{A}\mathrm{ReLU}\left(\tilde{A}XW^{\left(0\right)}\right)W^{\left(1\right)}\right)$$

where *W*^(0)^ ∈ *R*^*C*×*H*^ is the weight matrix from input to the hidden layer and *H* is the number of neurons in the hidden layer, and *W*^(1)^ ∈ *R*^*H*×*F*^ is the weight matrix from the hidden layer to the output layer. The neural network weights are trained using gradient descent. The cross-entropy is used as the loss function and its equation is as follows:


(8) $$L=-\left[y_{i}\log z_{i}+\left(1-y_{i}\right)\log\left(1-z_{i}\right)\right]$$

where *y*_*i*_ and *z*_*i*_ are the true and predicted labels of the nodes.

The specialty of our FBI-GCN lies in its ability to fuse biometrics information of individuals and the common characteristics of subjects when classifying PFPS diseases. As shown in Figure 1, when diagnosing PFPS, priority should be given to the subject whose population is with a high prevalence (e.g., female group, overweight group, etc.). The biosignal similarity of the subjects is also considered. These biometrics information is fused by weighting, and a graph is used to represent the association between subjects. The operation of GCN focuses on the biosignal information of the subject individuals with their common features. In this way, the classification of PFPS is made more reliable. When training the GCN, it focuses on the common characteristics of the subjects and the individual biosignal information so as to obtain a good classification capability.

## Results

### Experimental parameters and evaluation indexes

In the experiments, we chose the k-fold cross-validation (Refaeilzadeh et al., 2016) method to evaluate the generalization ability of our model with *k* = 5. To ensure the reliability of the results, each experiment was repeated 50 times independently and the mean and standard deviation were used for comparison. The evaluation indexes mainly include accuracy (ACC), sensitivity (also known as precision, represented by P), specificity (also known as recall, represented by R), and F1-Score (Cao et al., 2021). Their expressions are as follows:


(9) $$ACC=\frac{TP+TN}{TP+FP+FN+TN}$$

(10) $$P=\frac{TP}{TP+FN}$$

(11) $$R=\frac{TN}{TN+FP}$$

(12) $$F1-score=\frac{2\times P\times R}{P+R}$$

where *TP, FP, TN*, and *FN* represent the number of samples corresponding to the true positive, false positive, true negative, and false negative (positive is the subject predicted by the model as pain, and negative is the subject predicted as control) (Wu et al., 2022).

The parameters of our GCN model are set as follows: dropout rate: 0.5; learning rate: 0.01; epochs: 300; the number of hidden layers: 1; the number of nodes in the hidden layer: 64.

### Graph construction strategy

GCN aggregates node information according to the graph and the graph structure has a great impact on the performance of classification. In order to investigate the effect of different graph structures on the average classification accuracy, the following variations of the graph were analyzed: (1) select only the *Sim*(*V*_*i*_, *V*_*j*_) as the edge of the graph, (2) use equation (2) to fuse node feature similarity and subject demographic information, and (3) use γ to calculate the adjacency matrix of the graph.

The running information in the dataset, including kinematics of three joints and sEMG signals of seven muscles during running, was used as the node input. It is because the population with PFPS suffers from more pronounced pain during strenuous activities such as running (Glaviano et al., 2022). There are three options to use individual feature similarity to construct the graph: running, walking, and both.

**FIGURE 3:**
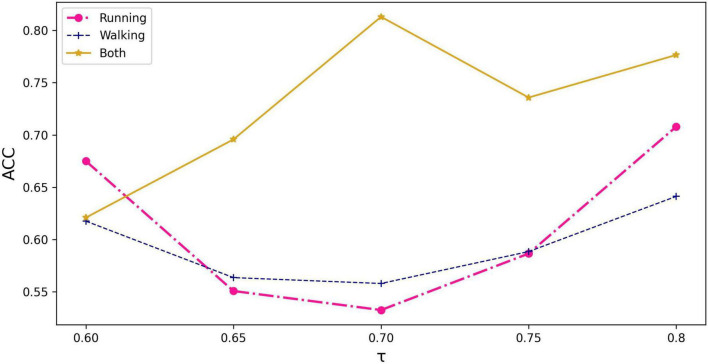
The impact of input information and threshold selection on classification performance expressed as accuracy. τ represents the threshold value being set in equation (3).

As can be seen from Figure 3, the graph constructed using all the information of running and walking with a threshold value of 0.70 has the highest accuracy. Based on this graph, demographic characteristics (gender, height, weight) were further added to compare their performance separately.

**FIGURE 4:**
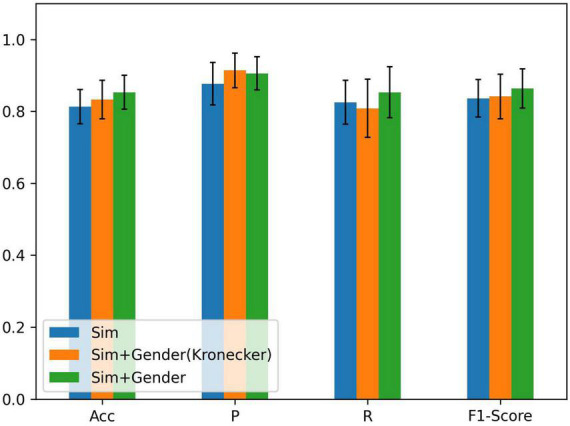
Comparison of different methods of fusing similarity and gender information: ACC = precision, P = sensitivity, and R = specificity; *Sim* denotes the similarity of node features, Sim + Gender (Kronecker) represents the use of the Kronecker delta function to express the function γ, and Sim + Gender denotes the use of equation (5) to express the weight matrix of the graph.

As there is a significant difference in the probability of PFPS between males and females (Rothermich et al., 2015), we set λ_1_ = 1 and λ_2_ = 2 in equation (5) to express gender differences and compared them using the Kronecker function. The input of the node still used all the biosignal information in running. The result is shown in Figure 4.

From Figure 4, we know that adding gender information to construct the graph has improved the classification performance and combining clinical knowledge gives better results. λ_*1*_ and λ_*2*_ should be set in the appropriate region. If the parameters were set too large, it results in other biometric information not being well represented. Relying on gender information cannot lead the model to better classification (refer to Table 2). If the parameter was set less than 1, it reduces the edge weight of the corresponding object, which is not conducive to the representation of prior knowledge. After repeated tests, λ_1_ = 1 and λ_2_ = 2 performed the best in the experiment.

**TABLE 2:** The effect of feature fusion on classification performance.

	Accuracy	Specificity	Sensitivity	F1-Score
Sim	0.8130 ± 0.048	0.8770 ± 0.059	0.8253 ± 0.061	0.8360 ± 0.052
Gender	0.4902 ± 0.079	0.6100 ± 0.092	0.5152 ± 0.100	0.5333 ± 0.085
Height	0.4573 ± 0.068	0.3198 ± 0.069	0.4373 ± 0.137	0.3501 ± 0.088
Mass	0.4872 ± 0.073	0.6420 ± 0.102	0.4357 ± 0.090	0.4943 ± 0.085
BMI	0.6046 ± 0.020	0.6207 ± 0.010	**0.9492 ± 0.036**	0.7410 ± 0.019
Sim + Gender	**0.8531 ± 0.047**	**0.9056 ± 0.047**	0.8532 ± 0.071	**0.8637 ± 0.055**
Sim + Height	0.7872 ± 0.056	0.8756 ± 0.054	0.7833 ± 0.078	0.8101 ± 0.060
Sim + Mass	0.8222 ± 0.044	0.7224 ± 0.089	0.8273 ± 0.084	0.7430 ± 0.072
Sim + BMI	0.8197 ± 0.042	0.8949 ± 0.055	0.8132 ± 0.054	0.8372 ± 0.048
Sim + Gender + Height + Mass	0.8198 ± 0.044	0.8934 ± 0.045	0.8148 ± 0.068	0.8381 ± 0.051
Sim + Gender + BMI	0.8454 ± 0.044	0.7750 ± 0.092	0.8076 ± 0.077	0.7652 ± 0.069

The bold values mean the best performing results in the experiment.

The dataset contains the information of the subjects’ height and weight in addition to their gender. To test the effect of using this information on PFPS classification, we tried to conduct the experiment using equation (4). A significant correlation between the weight of the subjects and gender was observed in Table 1 (the weight of males differed more from that of females). In order to better observe whether excess weight is correlated with PFPS, we chose to use BMI for further experiments.

From Table 2, we find that the accuracy of the classification is low when only the subjects’ gender, height, weight, or BMI is used to construct the graph, indicating that demographic information alone does not guarantee effective classification of PFPS. The performance of classification is improved when subjects’ demographic information fuses with the similarity of individual characteristics. Also, the classification accuracy after fusing BMI with similarity does not change much compared to the graphs constructed using only similarity. Combining the experimental results, we find that the graph constructed from similarity and gender showed the highest ACC and F1-scores among various methods.

### Selection of node input information

In the previous section, the graph construction strategy was discussed. This section will focus on the relationship between the node input information and the classification accuracy.

**FIGURE 5:**
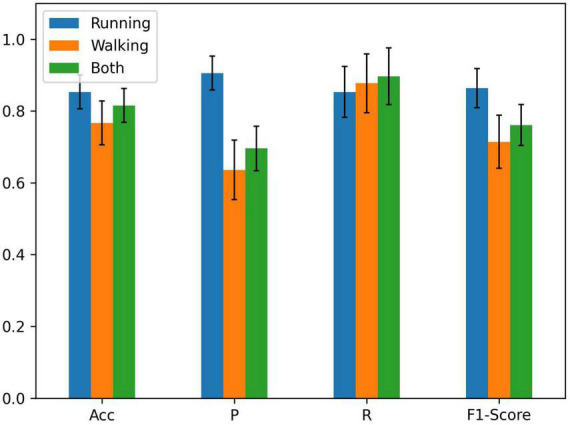
Comparison of running, walking, and both as node inputs.

Running and walking are dynamic muscle contractions and joint rotation (McGill, 2004), so the sEMG and joint angles are used as inputs for each node. The PFPS classification results using running, walking and both as input are explored separately in Figure 5. The graph used in the comparison experiment was the fusion of all biosignal in running and the gender information (Sim + Gender was mentioned in Figure 4, it had the best performance in the previous section). The results verify our initial conjecture that higher classification accuracy can be obtained by using the running data as the input.

### Feature optimization for input variables

To collect effective signals, the sensors measuring biological information are often high-precision instruments, so the experimental cost is relatively high. In some cases, irrelevant and misleading features may reduce the accuracy and speed of the prediction algorithm. On the other hand, the raw sEMG signals are redundant and sometimes contradictory (Oskoei et al., 2013). Therefore, it is meaningful to optimize the input features to improve the diagnostic performance of FBI-GCN.

**FIGURE 6:**
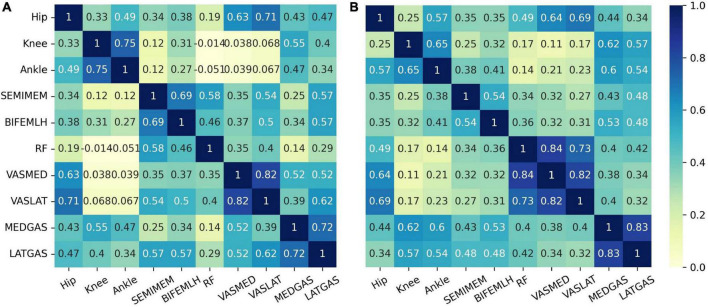
A heatmap of correlations between groups of variables. **(A)** the control group, **(B)** the PFPS pain group.

The related heatmap of the input variables’ intraclass Pearson correlation coefficient is shown in Figure 6. In the control group, the correlation is stronger for the knee and ankle joints among the three variables of joint kinematics. The correlations are strong for the same muscle groups, except for the rectus femoris of the quadriceps. However, correlations between the quadriceps and other muscle groups were significantly lower in the pain group than in the control group.

**FIGURE 7:**
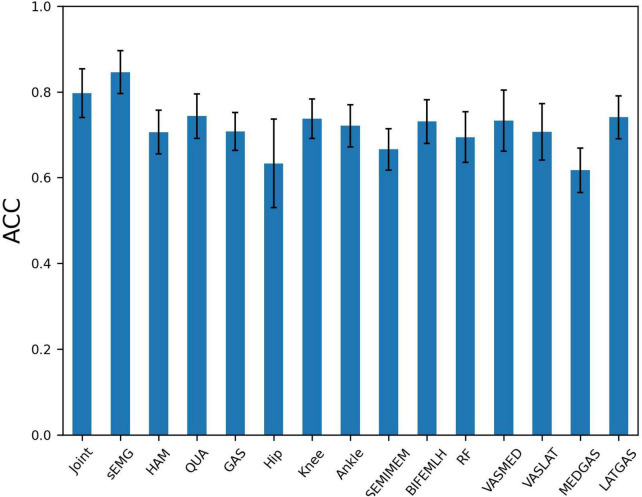
Patellofemoral pain syndrome (PFPS) classification accuracy for different node input information selection strategies: Joint, the joint angles of Hip Flexion, Knee Flexion, and Ankle Flexion when subjects in running; sEMG, the sEMG signals of 7 muscles in the dataset; HAM, hamstrings’ sEMG, including BIFEMLH and SEMIMEM; QUA, quadriceps’ sEMG, including RF, VASMED and VASLAT; GAS, gastrocnemius’ sEMG, including MEDGAS and LATGAS.

The presence of high correlation between variables of the same muscle group implies the possibility of redundant information. To further explore the influence of different node input information on the PFPS classification accuracy, different combinations of variables were used as the input to observe their effects on the PFPS classification performance. The experiment was conducted using the following three groups: (1) all sEMGs and all joint angles used as the input; (2) three muscle groups’ sEMG: hamstrings (HAM), quadriceps (QUA) and gastrocnemius (GAS) used as the input; and (3) individual joint angles and individual muscle sEMGs used as the input. The graph also used the fusion of all biosignal in running and the gender information. The experiment results are shown in Figure 7.

From Figure 7, we find that using all sEMG as node input has higher classification accuracy than using all joint angelas the input. Among the three muscle groups, using quadriceps’ sEMG as the input obtained the highest accuracy for PFPS classification. When using the three joint angles separately as the input, the combination of the knee and ankle joint angles results in a similar accuracy, but higher than the combination of the hip joint angles. In the seven muscles tested, using the combination of the lateral hamstrings, vastus medialis, and lateral gastrocnemius as inputs showed better accuracy than using the other muscles.

Based on the results in Figure 7, we selected the top five variables for testing in order of accuracy. These are LATGAS, Knee, BIFEMLH, VASMED, and Ankle, and they have exactly one of the sEMGs in each muscle group, as well as the joint kinematics of the knee and ankle joints. To deal with optimization problems, it is good to use metaheuristic algorithms based on swarm intelligence techniques (Li et al., 2022a). However, in this experiment, the variety of combinations is lower than the range of applicability of the heuristic intelligent algorithms. It is acceptable to use the exhaustive method for comparison. There are (2^5^–1) different combinations, we only show the cases with the highest accuracy using different numbers of variables, as is shown in Table 3.

**TABLE 3:** Variable combinations for node input.

Number of variables	LATGAS	Knee	BIFEMLH	VASMED	Ankle	ACC
1	1	0	0	0	0	0.7406 ± 0.050
2	1	0	1	0	0	0.8406 ± 0.037
3	1	0	1	1	0	0.8327 ± 0.046
4	1	0	1	1	1	**0.8422 ± 0.050**
5	1	1	1	1	1	0.8319 ± 0.052

The bold values mean the best performing results in the experiment.

As is shown in Table 3, when more than 1 variable is used, the accuracy is higher than 83%. We can also see from this table that the performance is the best when both variables LATGAS and BIFEMLH are used. Therefore, in the subsequent experiments, LATGAS and BIFEMLH will be used as the input information of the node.

In addition to the information of the node inputs, the graph structure also needs to be optimized. By comparing the two correlation heat maps in Figure 7, we find that the correlation between the three muscle groups in the PFPS pain group is weaker than that in the control group. In our FBI-GCN model, the graph focuses on the common biometric information of the subjects so that their association should be as differentiable as possible from the pain-free population. As the weakness of muscle groups above the knee has been well documented in young adults with PFPS (Crossley et al., 2016), it is speculated that using EMG signals from the quadriceps and other muscle for the composition could be a good representation.

Based on the discussions above, we try to use the following three kinds of combinations of features to optimize the graph construction: (1) the information from a single muscle group or a single joint kinematics; (2) the combination of only a few features; and (3) the data from one type of sensor, such as from only sEMG or joint kinematics.

**FIGURE 8:**
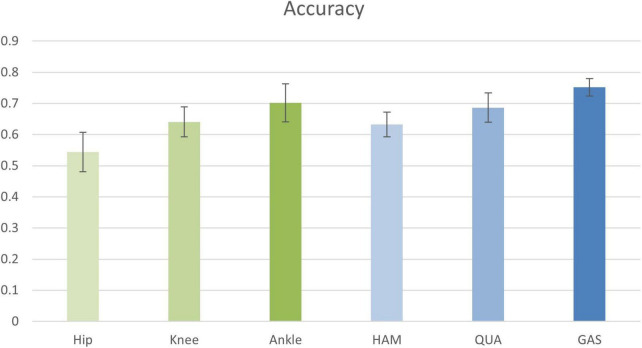
Classification accuracy of joint kinematics and muscle group conformation. The accuracy values shown in the figure are the result of the best performance of the post-configuration threshold selection between 0.7 and 0.85.

In the first experiment, the input information to the nodes was composed of the sEMG signals from LATGAS and BIFEMLH during running. The graphs were constructed according to the biosignal information of individual joint kinematics and single muscle groups. The results are shown in Figure 8, their prediction accuracy for PFPS ranged from 0.55 to 0.75, which is at a relatively low level.

It is impossible to get good classification results by relying only on a single bioinformatic composition of either joint dynamics or muscle groups. Therefore, we are going to test whether removing the EMG signal of a few muscle groups could contribute to the improvement of diagnostic accuracy. The results are shown in Table 4.

**TABLE 4:** Classification performance of FBI-GCN with removed composition variables.

Name of removed variables	Accuracy	Specificity	Sensitivity	F1-Score	τ	*M*_*h*_
Hip	0.8355 ± 0.047	0.7432 ± 0.087	0.8162 ± 0.081	0.7505 ± 0.078	0.75	None
Knee	0.8003 ± 0.038	0.8676 ± 0.039	0.8138 ± 0.048	0.8283 ± 0.036	0.8	Gender
Ankle	0.8252 ± 0.035	0.8896 ± 0.039	0.8358 ± 0.053	0.8460 ± 0.034	0.75	None
HAM	0.8393 ± 0.041	0.7700 ± 0.086	0.7799 ± 0.074	0.7493 ± 0.062	0.85	None
QUA	0.8309 ± 0.046	**0.9072 ± 0.040**	0.8258 ± 0.072	0.8455 ± 0.048	0.8	None
GAS	**0.9245 ± 0.034**	0.8635 ± 0.072	**0.9419 ± 0.074**	**0.8836 ± 0.064**	0.75	None
Hip and GAS	0.8217 ± 0.051	0.8885 ± 0.043	0.8359 ± 0.070	0.8435 ± 0.052	0.75	None
Knee and GAS	0.7734 ± 0.049	0.8726 ± 0.048	0.7583 ± 0.077	0.7929 ± 0.058	0.75	Gender
Ankle and GAS	0.8029 ± 0.040	0.6857 ± 0.085	0.8079 ± 0.090	0.7168 ± 0.075	0.75	Gender
HAM and GAS	0.8627 ± 0.047	0.8113 ± 0.089	0.8440 ± 0.072	0.7973 ± 0.068	0.85	Gender
QUA and GAS	0.6798 ± 0.034	0.8022 ± 0.044	0.8021 ± 0.077	0.7123 ± 0.044	0.85	Gender
Hip, HAM and GAS	0.799 ± 0.044	0.7162 ± 0.097	0.7277 ± 0.090	0.6856 ± 0.079	0.8	None

The bold values mean the best performing results in the experiment.

As can be seen from Table 4, the best classification performance was obtained from our FBI-GCN model when the biosignal from the gastrocnemius muscle was removed (accuracy = 0.9245 ± 0.034, sensitivity = 0.9419 ± 0.074). If only the biosignal of joint kinematics and quadriceps were used, it achieved a better performance (accuracy = 0.8627 ± 0.047) than when all information was retained.

The final experiment was conducted by removing some variables separately, using the same type of sensor. Results are shown in Table 5. We can see from the above table that using only joint kinematic information as the input combination did not ensure correct classification of PFPS. Using the sEMG of the quadriceps and hamstrings as the input combination could result in good classification performance with 40% fewer measured variables.

**TABLE 5:** Classification performance of FBI-GCN by removing some variables separately according to different outline quantities.

Name of variables	Accuracy	Specificity	Sensitivity	F1-Score	τ	*M*_*h*_
Hip, Knee and Ankle	0.5657 ± 0.057	0.4337 ± 0.068	0.5974 ± 0.1223	0.4738 ± 0.079	0.75	Gender
HAM, QUA and GAS	0.8010 ± 0.051	**0.8517 ± 0.052**	0.8256 ± 0.074	**0.8258 ± 0.056**	0.75	Gender
HAM and QUA	**0.8806 ± 0.031**	0.8180 ± 0.072	**0.8791 ± 0.072**	0.8235 ± 0.064	0.8	None
HAM and GAS	0.6800 ± 0.047	0.7739 ± 0.049	0.7118 ± 0.061	0.7236 ± 0.045	0.85	Gender
QUA and GAS	0.7417 ± 0.046	0.8275 ± 0.054	0.7585 ± 0.060	0.7710 ± 0.051	0.8	None

The bold values mean the best performing results in the experiment.

### Comparison with other methods

**FIGURE 9:**
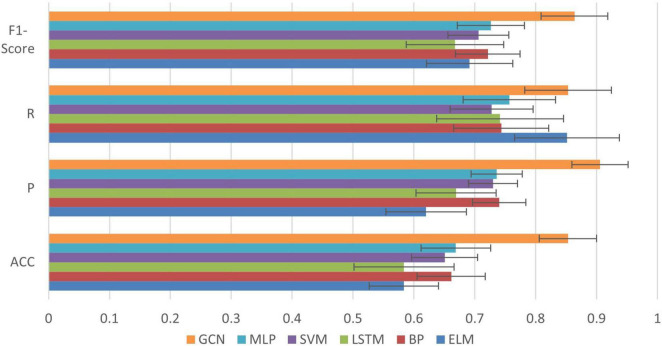
Comparison of classification results between GCN and other common algorithms.

In order to further confirm the performance of our method, we compare it against the following five methods: Extreme Learning Machines (ELM), Support Vector Machine (SVM), Multilayer Perceptron (MLP), Back Propagation Neural Network (BP) and Long Short-Term Memory (LSTM) (Shi et al., 2019; Shi et al., 2021b). Among them, the kernel function of the SVM is linear, the *C*-value is set to 0.04. In the MLP, there is one hidden layer with 150 neurons trained for 3000 iterations (Shi et al., 2019). The ELM uses one hidden layer with 174 neurons. There is also one hidden layer in the BP network, and the number of neurons in the hidden layer is 37. The LSTM uses 32 neurons and the number of iterations is 3000. The learning rate for all of these models is set to 0.01 (Shi et al., 2021b). Each experiment was performed with a fivefold cross validation method and the experiment was repeated 50 times to work out the mean and the standard deviation. The input of all algorithms was biosignal information during running (three joint kinematics and sEMG of seven muscles). The graph in GCN was used the same input to construct. The results are shown in Figure 9.

**FIGURE 10:**
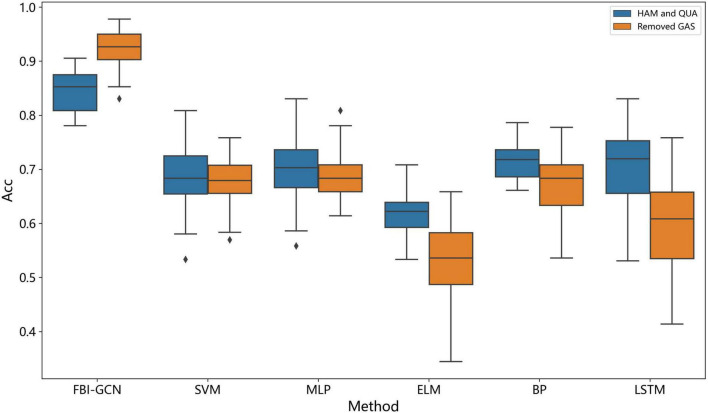
Comparison of the accuracy after input optimization.

We can see from Figure 9 that compared with the other algorithms, our method achieved the best performance in all evaluation indexes with lower deviation. The optimized performance of FBI-GCN is more prominent, and the comparison experiment is shown in Figure 10. We chose the cases of “Removed GAS” (listed in Table 4) and “HAM and QUA” (listed in Table 5), two different input information of FBI-GCN, as control tests. The input of the other algorithms, respectively, used the same information as the two optimization case.

We can see from Figure 10 that the combination of input variables has a significant impact on the performance of the classification. The selection of input variables, combined with the method of biometric fusion, can obtain significantly higher classification performance than using common machine learning.

## Discussion

We have proposed an end-to-end GCN method as an auxiliary tool for PFPS diagnosis. By optimizing the sparse map based on clinical knowledge, a more accurate diagnostic result can be obtained. Extensive experiments on a public PFPS dataset have been carried out to verify the effect of our model and obtained an average accuracy of 0.8531, specificity of 0.9056, sensitivity of 0.8532, and F1-Score of 0.8637. The highest classification accuracy (mean accuracy = 0.9245 ± 0.034) was obtained after optimal selection of input variables and a higher accuracy (mean accuracy = 0.8802 ± 0.035) was also obtained after 40% of the measured variables had been removed.

Previous studies showed that anterior knee pain elicited during a squatting maneuver, which is evident in 80% of those who are positive in the test (Crossley et al., 2016). The specificity of palpation using the patellar margin is evident in 71–75% of patients (Nunes et al., 2013). There are also tests with limited diagnostic usefulness, such as patella grinding and fear tests (Doberstein et al., 2008). Compared with conventional PFPS clinical tests, the overall performance of our proposed complementary diagnostic method is proved better.

From the above experiments, the most important parts that affect the performance of GCN classification are the overall graph selection and input information. Therefore, it is crucial to design an effective graph. The results showed that adding redundant biometric information can lead to a severe decrease in classification performance. In contrast, accurate clinical guidance information (gender) can significantly improve diagnostic performance. In the experiments, the accuracy of the classification did not change much after the node similarity fused the information of BMI, because there is no significant correlation between BMI and PFPS in this dataset. The BMI of the subjects was basically in normal condition (see Table 1) and there were no obese or overweight subjects, so the classification performance could not be improved by adding the information of BMI in the composition. This situation was also observed by Hart et al. (2017), which showed that higher BMI was present in PFPS but not in the adolescents with PFPS. Since GCN only works with structurally fixed graphs, although replacing a certain node does not have a significant impact on the classification performance, the model needs to be retrained based on the modified graph. Therefore, it is more cost effective to apply the auxiliary detection method to group detection than to single detection.

Another important factor is the node input information. It is generally necessary to reconstruct the data and extract the features (Bi et al., 2019). Ideally, it is better to use an end-to-end strategy when the desired results are obtained using the raw data as the input to the model. This is the main advantage of deep learning and one of the reasons why people are keen to use it to solve real-world problems. The GCN diagnostic method does not require the use of biomechanical models along with complex input variables, but only a small amount number of joint angles and the sEMG. These data can be collected in everyday environments without any physical impact on the subject. The method is more objective, convenient and versatile than traditional testing ones.

In addition, in the optimization experiment of node input, using only the sEMG of lateral hamstrings and lateral gastrocnemius as node input information can achieve better classification performance. During exercise, early activation of the lateral hamstrings relative to the medial hamstrings plays a role in PFPS (Karagözoðlu Coşkunsu et al., 2022). Therefore, as the input variable of the node, the sEMG signal of the lateral hamstrings plays an important role in the diagnosis of PFPS. In the experiment of optimizing the structure of the observation graph, using only the quadriceps with other muscle groups or joint kinematics can obtain better classification. Other researches have shown that the decrease of quadriceps strength is an important factor in PFPS (Vora et al., 2017; Malmir et al., 2022). It is known that the identified positive potential risk factors included: gastrocnemius, hamstring or quadriceps tightness, deficient hamstring or quadriceps strength, among others (Waryasz and McDermott, 2008; Petersen et al., 2014). In terms of joint kinematics, Lankhorst et al. (2012) comprehensively evaluated the results of previous related studies and found that lower knee extension strength was a risk factor for PFPS. Bolgla et al. (2011) indicated that the range of motion of the ankle dorsiflexors was reduced in patients with PFPS. That is, the knee and ankle information of PFPS patients will be more important for the diagnosis. Our experimental results shown in Table 4 also observed these relationships. Relative to the knee and ankle joints, the removal of information about the hip joint has less impact on the classification performance.

## Conclusion

In this paper, we proposed a GCN-based disease diagnosis method, FBI-GCN, which focuses on both individual biosignal information and common features of patients by fusing biometric information for improving the diagnostic accuracy. After optimization of node inputs and graph structure, better diagnostic performance was obtained with 40% fewer measured variables. Our future work would mainly focus on the following three aspects. One is to validate on a larger dataset and classify various gait diseases. The second is to further optimize the model to improve the accuracy in PFPS detection. The third is to further explore the hidden relationship between PFPS and human biosignal.

## Data availability statement

Publicly available datasets were analyzed in this study. This data can be found here: https://www.sciencedirect.com/science/article/pii/S0021929009000396?via%3Dihub.

## Author contributions

BX, YO, GM, and YX conceived the layout, the rationale, and the plan of this manuscript. BX, YO, and YC wrote the first draft of the manuscript. MD, BL, and YX edited the manuscript. All authors contributed to the article and approved the submitted version.

## Footnotes

1
https://www.sciencedirect.com/science/article/pii/S0021929009000396?via%3Dihub
